# Evaluating ECM stiffness and liver cancer radiation response via shear-wave elasticity in 3D culture models

**DOI:** 10.1186/s13014-024-02513-7

**Published:** 2024-09-27

**Authors:** Shao-Lun Lu, Yu Pei, Wei-Wen Liu, Kun Han, Jason Chia-Hsien Cheng, Pai-Chi Li

**Affiliations:** 1 Department of Radiation Oncology, National Taiwan University Cancer Center, Taipei, Taiwan; 2 Graduate Institute of Oncology, National Taiwan University College of Medicine, Taipei, Taiwan; 3 Graduate Institute of Biomedical Electronics and Bioinformatics, National Taiwan University, Taipei, Taiwan; 4 Graduate of Institute of Oral Biology, National Taiwan University College of Medicine, Taipei, Taiwan; 5 Division of Radiation Oncology, National Taiwan University Hospital, Taipei, Taiwan; 6 Department of Electrical Engineering, National Taiwan University, Taipei, Taiwan

**Keywords:** Tumor microenvironment, Radiosensitivity, Extracellular matrix stiffness, Three-dimensional culture, Lysyl oxidase, Sonoporation

## Abstract

**Background:**

The stiffness of the tumor microenvironment (TME) directly influences cellular behaviors. Radiotherapy (RT) is a common treatment for solid tumors, but the TME can impact its efficacy. In the case of liver cancer, clinical observations have shown that tumors within a cirrhotic, stiffer background respond less to RT, suggesting that the extracellular matrix (ECM) stiffness plays a critical role in the development of radioresistance.

**Methods:**

This study explored the effects of ECM stiffness and the inhibition of lysyl oxidase (LOX) isoenzymes on the radiation response of liver cancer in a millimeter-sized three-dimensional (3D) culture. We constructed a cube-shaped ECM-based millimeter-sized hydrogel containing Huh7 human liver cancer cells. By modulating the collagen concentration, we produced two groups of samples with different ECM stiffnesses to mimic the clinical scenarios of normal and cirrhotic livers. We used a single-transducer system for shear-wave-based elasticity measurement, to derive Young’s modulus of the 3D cell culture to investigate how the ECM stiffness affects radiosensitivity. This is the first demonstration of a workflow for assessing radiation-induced response in a millimeter-sized 3D culture.

**Results:**

Increased ECM stiffness was associated with a decreased radiation response. Moreover, sonoporation-assisted LOX inhibition with BAPN (β-aminopropionitrile monofumarate) significantly decreased the initial ECM stiffness and increased RT-induced cell death. Inhibition of LOX was particularly effective in reducing ECM stiffness in stiffer matrices. Combining LOX inhibition with RT markedly increased radiation-induced DNA damage in cirrhotic liver cancer cells, enhancing their response to radiation. Furthermore, LOX inhibition can be combined with sonoporation to overcome stiffness-related radioresistance, potentially leading to better treatment outcomes for patients with liver cancer.

**Conclusions:**

The findings underscore the significant influence of ECM stiffness on liver cancer’s response to radiation. Sonoporation-aided LOX inhibition emerges as a promising strategy to mitigate stiffness-related resistance, offering potential improvements in liver cancer treatment outcomes.

**Supplementary Information:**

The online version contains supplementary material available at 10.1186/s13014-024-02513-7.

## Introduction

The success of radiotherapy (RT) in treating tumors is significantly influenced by certain characteristics of the tumor microenvironment (TME) [1, 2], including the stiffness of the extracellular matrix (ECM). Clinical observations have consistently demonstrated that liver tumors developing within a cirrhotic, stiffer background respond less to RT [3]. This clinical finding highlights the critical role of ECM stiffness in the development of radioresistance and underscores the need to understand the underlying mechanisms.

The TME stiffness is determined by the composition and organization of the ECM [4], which provides structural support and influences cellular behaviors. Studies have shown that a stiffer ECM promotes the progression and invasion of cancer as well as therapy resistance [5]. In the context of RT, the impact of ECM stiffness on treatment response is receiving increasing attention. However, liver cancer within a cirrhotic background presents a unique challenge due to the pronounced stiffness associated with liver fibrosis.

Liver fibrosis is characterized by excessive collagen deposition and ECM remodeling and is a common consequence of chronic liver diseases such as cirrhosis [6]. Specifically, the cirrhotic ECM is rich in collagen types I and III, fibronectin, and laminin [7]. This fibrotic ECM creates a stiffer microenvironment that significantly impacts cellular behaviors and contributes to the development and progression of liver cancer [8]. Lysyl oxidase (LOX) isoenzymes are dependent on copper and operate extracellularly to facilitate the cross-linking of collagens within the ECM in a fibrotic liver [9]. Understanding the mechanisms underlying the worse response of liver tumors within a cirrhotic, stiffer background to RT is of paramount importance for improving treatment outcomes.

The clinical role of radiation therapy for liver cancer has advanced with the development of techniques like stereotactic body radiation therapy (SBRT) and proton beam therapy, making it a valuable option for inoperable cases or for those with limited liver reserve [10]. However, patients with advanced hepatic cirrhosis experience worse outcomes after RT compared to those with less severe cirrhosis [11, 12]. Radiation-induced liver injury presents a significant challenge for liver cancer RT, and achieving optimal tumor control in a cirrhotic liver remains difficult. The current understanding of stiffness-related radioresistance is mainly based on experiments utilizing polyacrylamide-based hydrogels. DNA repair has been demonstrated to be affected by ECM stiffness, with low elasticity impairing ubiquitin conjugation at the site of double-strand breaks (DSB) [13]. However, most investigations of three-dimensional (3D) cultures have involved hydrogels thinner than 100 μm. It has been shown that cell mechanosensing responses are dominated by the stiffness of the collagen coupled directly to the polyacrylamide background rather than to the surrounding ECM [14]. The smaller dimensions and anisotropic stiffness between the ECM and the backbone hydrogel mean that these in vitro studies are not realistic representations of the in vivo conditions [14]. A 3D culture with an isotropic stiffness would be a better platform for investigating the association between ECM stiffness and radiosensitivity.

We have developed a platform for shear-wave (SW)-based elasticity measurement with a single ultrasound transducer for investigating millimeter-sized biomaterial samples [15]. The stiffness of the ECM could be probed by measuring the SW speed (SWS) within the defined volume. The platform was used to investigate 3D cultures of liver cancer cells. This study aimed to determine, for the first time, the impact of the TME stiffness on the radiation response of human liver cancer in a millimeter-sized 3D culture. Notably, unlike the two-dimensional clonogenic assay, in which the numbers of colonies are counted for the successful cell division after RT, there is no standard workflow for assessing the radiosensitivity in such a millimeter-sized 3D culture.

By investigating the impact of ECM stiffness on radiation response using a millimeter-sized 3D culture model, this study aimed to elucidate the factors contributing to radioresistance in liver cancer. We additionally explored novel strategies such as LOX inhibition (LI) and sonoporation for overcoming stiffness-related radioresistance and enhancing the efficacy of RT in liver cancer patients. Through these investigations we hoped to facilitate more effective treatment approaches and improved clinical outcomes in the management of liver cancer.

## Methods and materials

### Hepatocellular carcinoma cell lines

The human hepatocellular carcinoma cell line Huh7 was obtained from the JCRB cell bank (Okayama, Japan). Cells were cultured in DMEM, supplemented with 10% fetal bovine serum and 50 U/mL penicillin/streptomycin, and kept at 37 °C in a humidified atmosphere of 5% CO_2_ and 95% air. To ensure the integrity and validity of our cell cultures, we performed regular mycoplasma contamination checks before each batch of experiments.

### SW-based elasticity measurements

The principle of the SW-based elasticity measurement has been reported previously [15]. Specifically, a custom-made single-element 20 MHz focused ultrasound transducer was securely installed on a designed fixture mounted on the sample container. The single-element transducer both generates and detects SWs. The elasticity images were produced using a one-dimensional autocorrelation algorithm at a sampling frequency of 5 kHz. The one-way distances between the focal point of the ultrasound transducer and the side boundaries were fixed at 3.0, 6.2, 9.2, and 12.0 mm. With known propagation distances, the SWS in the sample can be obtained by averaging the two-way propagation distances for the arrival times of the four SWs; that is,

$$\:SWS=\frac{\sum\:_{i}^{4}\frac{{d}_{i}}{{t}_{i}}}{4}.$$

**Fig. 1:**
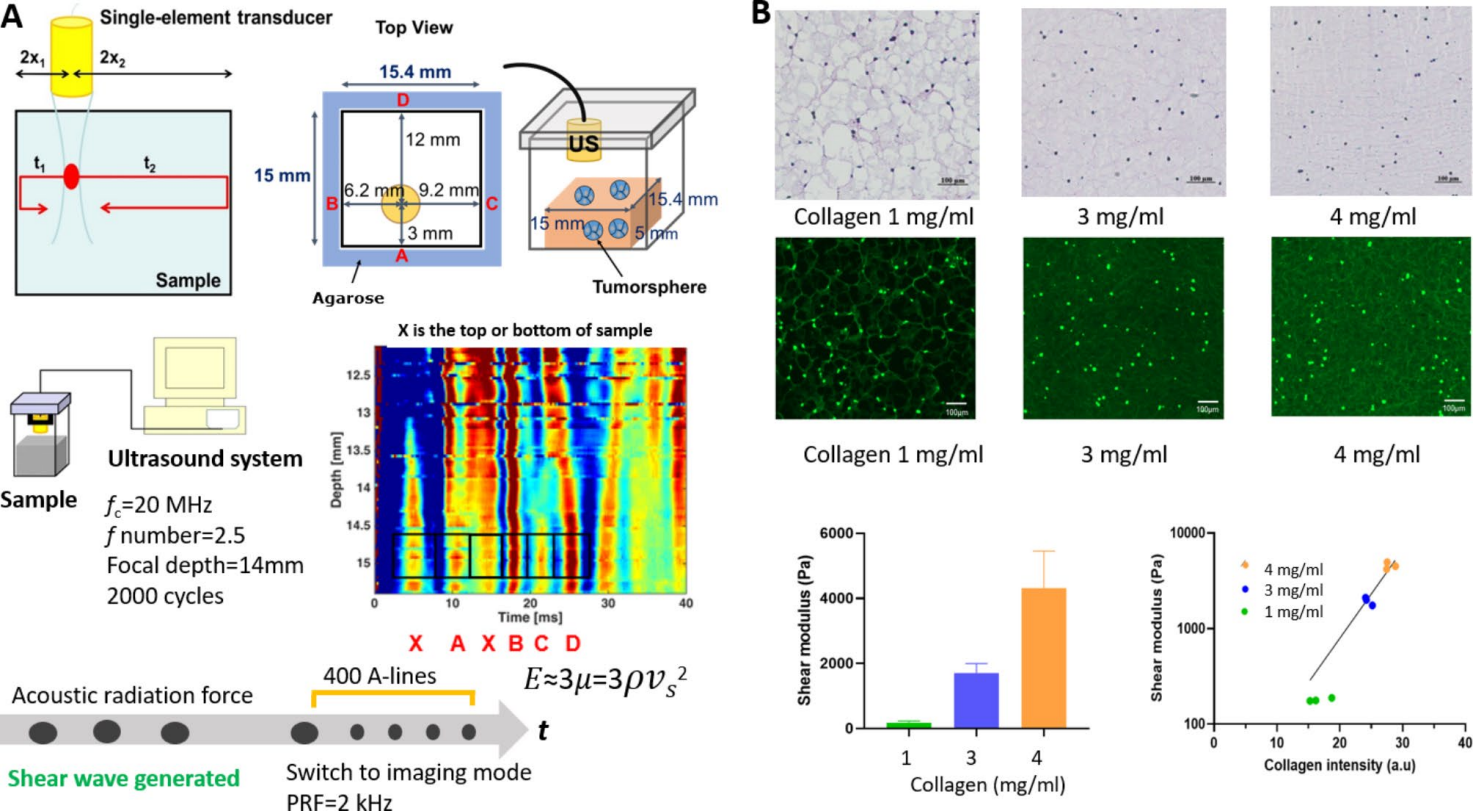
Shear-wave (SW)-based elasticity measurement system and correlation between collagen concentration and SW speed (SWS). (**A**) Schematic depiction of the single-element ultrasound transducer for SW-based elasticity measurement, showing the positioning of the ultrasound transducer relative to the sample side boundaries. The stiffness of the extracellular matrix (ECM)-based culture was determined by calculating the SWS for four reflected SWs. (**B**) Correlation between the concentration of collagen type I and SWS, showing representative hematoxylin and eosin images and fluorescence images from the three culture groups with different collagen type I concentrations. The scale bar indicates 100 μm. The bar diagram presents the mean and standard deviation (SD) values of the shear modulus. The line graph is a semilogarithmic plot showing the relationship between collagen intensity and shear modulus as derived from nine independent measurements (*n* = 3 in each group). PRF, pulse repetition frequency

Young’s modulus of the biomaterial could be derived as 𝐸≈3𝜇=3𝜌𝑣_𝑠_^2^, where *E* is Young’s modulus, 𝜇 is shear modulus, 𝜌 is sample density, and 𝑣_𝑠_ is the velocity of the SW. The platform measures the stiffness at the macroscale (on the order of millimeters). Figure 1A shows a schematic of the platform as well as the principle of deriving the stiffness from calculating the SWS of reflected SW.

### 3D cell culture preparation

As illustrated in Fig. 1A, a cuboid sample with dimensions of 15 mm × 15.4 mm × 5 mm (width × length × height) was surrounded by an agarose structure made with 0.5% agarose. The agarose structure supports the sample during polymerization and also serves as the SW reflector. The 3D culture system was established using a composite ECM of Matrigel and collagen type I to mimic the biochemical environment of the liver. Matrigel is a reconstituted basement membrane preparation extracted from mouse sarcoma, rich in laminin, collagen IV, heparan sulfate proteoglycans, entactin/nidogen, and growth factors [16]. Collagen type I, a major structural protein in the liver ECM, was added to this mixture to simulate the increased collagen content seen in cirrhotic liver tissue. We initially prepared three distinct sets of cell cultures by varying the concentrations of collagen type I (Corning, New York, USA): 1, 3, and 4 mg/ml. Across these groups, we consistently used Matrigel (Corning) at a concentration of 5 mg/ml.

The matrix gel was prepared by first mixing collagen type I with a pH neutralizing buffer containing 1 M NaOH, PBS, 0.9 mM CaCl_2_, 0.5 mM MgCl_2_, and sterilized distilled water. The Huh7 cancer cells were trypsinized from a culture plate and resuspended at 3.4 × 10^6^ cells/ml in serum-free tumorsphere medium (TSM) before being carefully mixed with the Matrigel matrix. The mixture of Matrigel and Huh7 cells was then added to the collagen mixture and thoroughly combined. Finally, we added biocompatible silicon dioxide at a mass concentration of 0.18% to the gel mixture to act as ultrasound scatterers. The matrix gel was prepared on ice to avoid polymerization. The matrix gel was carefully loaded into the cubic void in the agarose structure. The side channels were filled with 200 µl of TSM to provide sufficient moisture to the gel during the polymerization process. The entire cell culture sample was then incubated at 37 °C for 150 min. After polymerization, 7 ml of TSM was added to the top of the culture and the side channels. The medium was replaced every 24 h to ensure adequate exchanges of nutrients and waste products.

### X-ray irradiation and SW-based elasticity measurements

**Fig. 2:**
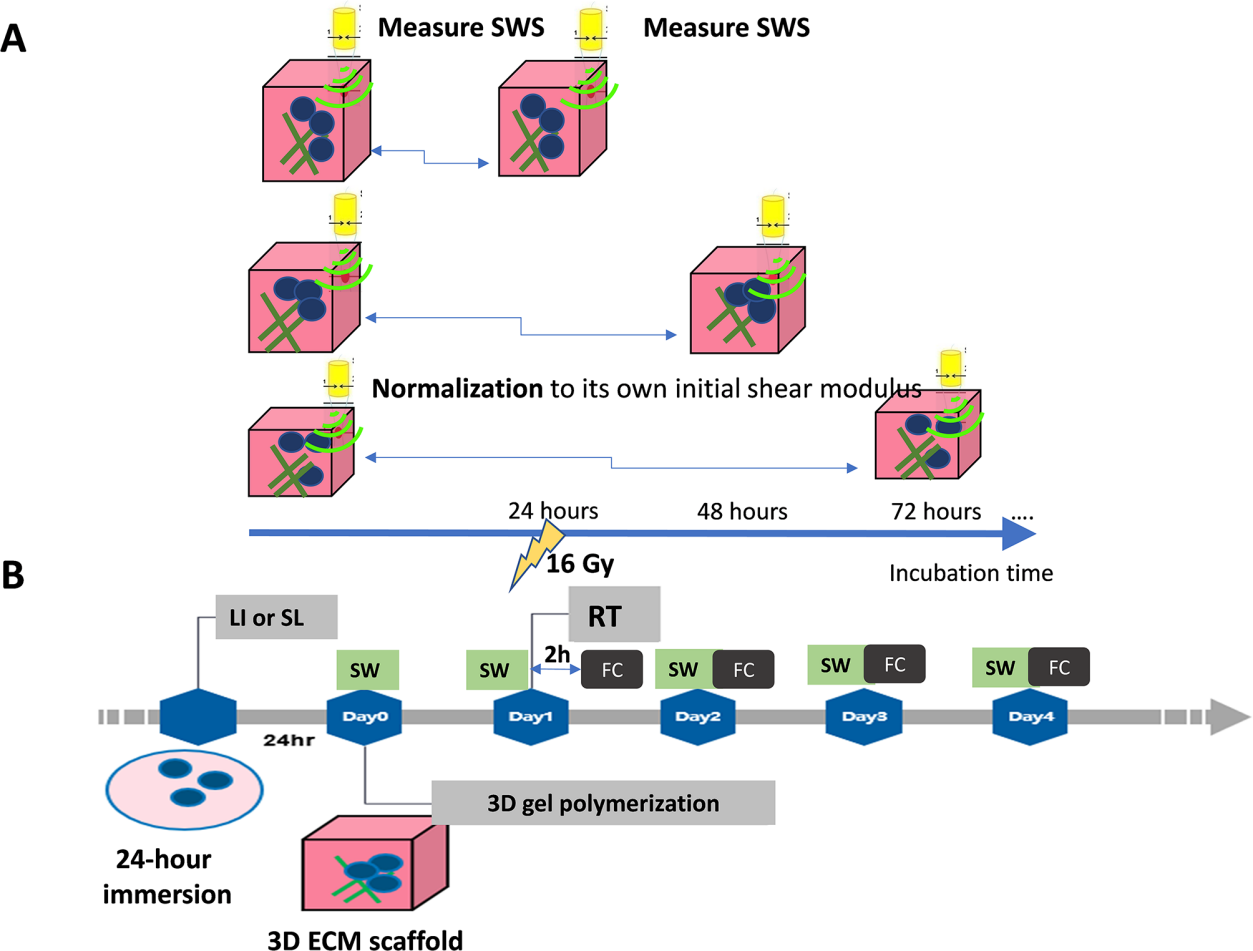
Experimental design and lysyl oxidase (LOX) inhibition (LI) combined with sonoporation (SL). (**A**) Schematic representation of the experimental design for monitoring the temporal dynamics of sample stiffness following radiotherapy (RT). The shear modulus was derived by calculating shear wave speed (SWS) to measure elasticity every 24 h. Stiffness values were normalized to the initial measurements made at the time of gel polymerization. (**B**) LI or SL was applied prior to gel polymerization and RT. The flow cytometry assessments for γ-H2AX, cleaved PARP1, and 7-AAD were conducted 2 h (2 h) after RT and every 24 h along with the measurement of SWS. SW: shear wave speed measurement, FC: flow cytometry assessment

The culture samples were irradiated with 225-kVp ionizing photons at 16 Gy (SmART, Precision X-Ray, Connecticut, USA) at 24 h after polymerization. RT with 16-Gy photons was administered 24 h after completing gel polymerization to allow the cells time to interact with their ECM. Elasticity measurements by calculating SWS were performed immediately after gel polymerization and then every 24 h up to 96 h. Considering the inherent stiffness variations within each gel despite using standard procedures for gel mixing and polymerization, we calculated how the shear modulus of each gel changed with the incubation period. We then normalized them to their initial values for longitudinal observations of stiffness dynamics (Fig. 2A).

### LI and sonoporation

Cross-linking of the ECM in the 3D culture was inhibited using 3-aminopropionitrile fumarate salt (BAPN; sc-214124, Santa Cruz Biotechnology, Dallas, USA), which is an irreversible LOX inhibitor. In the case of cultures subjected to LI, Huh7 cells were treated with 2 mM BAPN at 24 h prior to their incorporation into the collagen type I/Matrigel mixture (as shown in Fig. 2B). TSM containing 2 mM BAPN was utilized throughout the incubation period.

Sonoporation was applied using a 1-MHz focused single-element transducer in conjunction with microbubbles (MBs) for acoustic stimulation [17]. This procedure also involved the addition of 2 mM BAPN to the MB solution for a 5-minute sonoporation treatment of Huh7 cells before a 24-hour immersion period. The sonoporation was carried out at a pulse repetition frequency of 100 Hz with a pulse duration of 30 µs.

.

### Cancer cell recovery and flow cytometry assessments

The present noninvasive measurements allowed longitudinal biological investigations of the 3D culture after elasticity measurements. The protocol for viable cell recovery is described as follows: The sample was first carefully removed from the agarose supports and then chemically digested in 4 ml of calcium chloride aqueous solution (1 M) with 0.65 U/ml collagenase I (Gibco 17100-017, Thermo Fisher Scientific, Waltham, USA) and 36.4 U/ml dispase II (Gibco 27250018) for 15 min at 37 °C. The solution was then pipetted for 3 min to induce mechanical dissociation. After assessing the cell count and viability using trypan blue staining, the solution was centrifuged at 1600 rpm with 305 g for 3 min. The pellet was then resuspended in a single-cell solution before further flow cytometry analyses (BD FACSCalibur, Franklin Lakes, USA) within 2 h after RT.

γ-H2AX is an indicator of DNA DSB [18]. By measuring the signal intensity of γ-H2AX using flow cytometry, we evaluated the influence of cancer cells receiving RT in the 3D gel. The cancer cells were washed with PBS, fixed with 75% alcohol solution at − 20 °C for 30 min, and then permeabilized with 0.1% Triton X-100 in PBS solution. The antibody PE-γ-H2AX[Ser139] (Invitrogen 12-9865-42, Thermo Fisher Scientific) or FITC-anti-PARP1 (Invitrogen 53-6668-42, Thermo Fisher Scientific) was added at a ratio of 1000:5. In addition to the fixed sample assessed using γ-H2AX and PARP1 (cleaved form), fresh samples were stained with 2.5 µg/ml 7-aminoactinomycin D (7-AAD; Invitrogen 00-6993-50, Thermo Fisher Scientific) in flow cytometry to detect dead cells [19]. After a reaction interval of 30 min in the dark at 4 °C, the cell suspension was analyzed using a flow cytometer. The excitation wavelength was set at 488 nm, and emission filters of 515–545 nm, 564–606 nm, and > 650 nm were respectively used for detecting γ-H2AX, cleaved PARP1 (cPARP1), and 7-AAD. The signals were collected from 30,000 cell events for subsequent analyses.

### Hematoxylin and eosin staining, immunofluorescence, and fiber density assessments

Following the shear-wave-based elasticity measurements, the gel was fixed and then embedded using a mixture of 4% paraformaldehyde and 25% glutaraldehyde (Sigma-Aldrich, St. Louis, USA) at a ratio of 24:1. This embedding process was performed at room temperature for 6 h. Uniform horizontal 10-µm-thick sections subsequently obtained from all gel samples were then transferred for further processing and staining. Specifically, the sections received hematoxylin and eosin staining, immunostaining with a collagen type I antibody (GTX26308, GeneTex, Irvine, USA), and counterstaining with Hoechst 33,258 (Thermo Fisher Scientific). Images of the entire gel sections were captured using a microscopy system (AxioImager M1, Zeiss, Oberkochen, Germany). The fluorescence intensity of collagen type I was quantified using 8-bit measurements with ImageJ software. This analysis allowed for the calculation of pixel intensities across the entire section.

### Statistical analysis

To analyze the longitudinal dynamics of 3D sample stiffness, SWS, RT-induced γ-H2AX, 7-AAD, and cPARP1, we employed two-way repeated measures ANOVA using GraphPad Prism V9.0. For the multiple comparisons across different time points and treatment groups, we applied the Šídák correction to control the family-wise error rate. Additionally, we validated the findings by restricting the false discovery rate at 0.05 using the two-stage step-up method of Benjamini, Krieger, and Yekutieli.

## Results

### 3D culture grading by ECM stiffness

One of the objectives of this study was to build a 3D cell culture platform with a scale close to that of live animal experiments to investigate how modulating the ECM affected cancer behaviors. There are various reports in the literature on animal models with different severities of cirrhotic liver background that have been utilized to produce tumors with different stiffnesses [20]. The present study controlled the collagen composition of the matrix to construct samples with three elasticities (Fig. 1B). The mean shear moduli calculated for 3D cell cultures were 184, 1705, and 4318 Pa. Figure 1B shows the H&E and fluorescence images of the collagen type I fibers of the cultures with the three stiffnesses. A higher collagen concentration led to an increase in shear modulus, which showed an exponential correlation with the fluorescent intensity of the collagen. To specifically explore the influence of ECM stiffness on radiosensitivity, we chose cultures with collagen concentrations of 3 and 4 mg/ml, which were designated as normal and cirrhotic, respectively. These concentrations roughly mirror the stiffnesses found in healthy and cirrhotic microenvironments, corresponding to shear moduli of approximately 1.5 and 4.5 kPa, respectively. These choices were motivated by the clinical relevance of these stiffnesses to the human liver [21, 22].

### Temporal changes in stiffness following RT

**Fig. 3:**
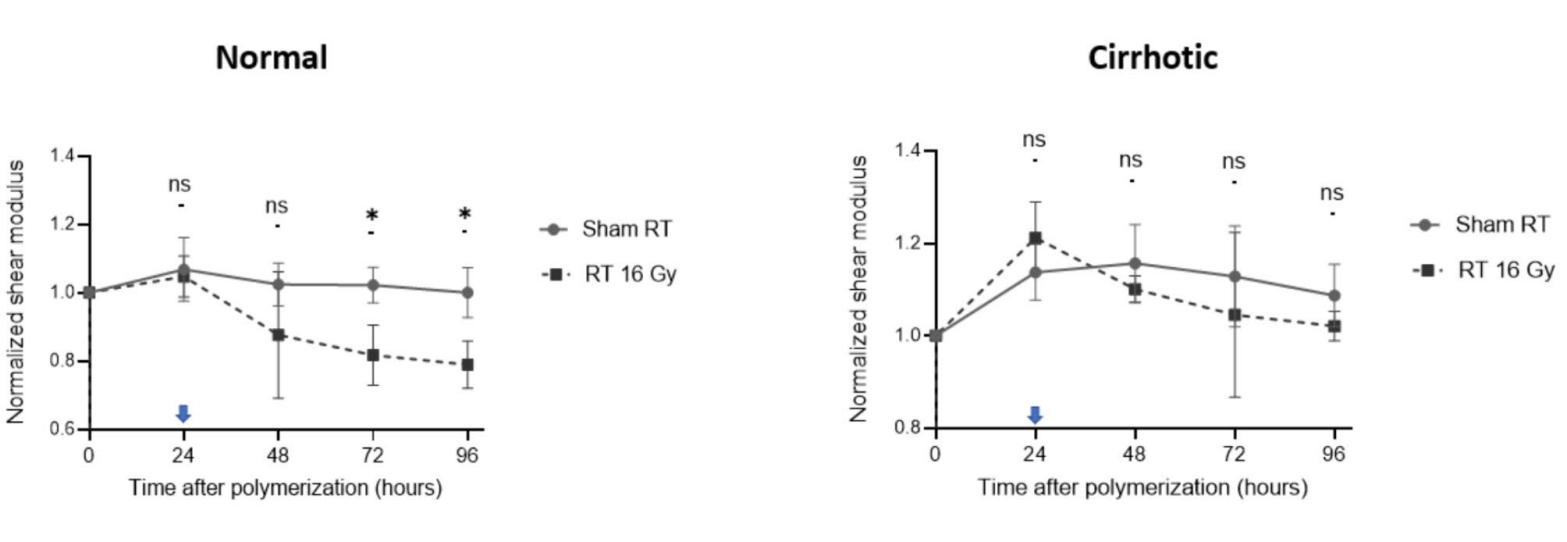
Normalized shear modulus over time in normal and cirrhotic matrix with and without radiation treatment. Temporal dynamics of the normalized shear modulus in Huh7-cell-containing gels with varying matrix stiffnesses after RT. Arrows indicate when RT at 16 Gy was applied. Data are mean and SD values from three independent experiments. * Indicates *p* < 0.05 and “ns” indicates non-significant differences between sham and 16 Gy irradiation after applying the Šídák correction for multiple comparisons at each time point among RT treatment groups

**Fig. 4:**
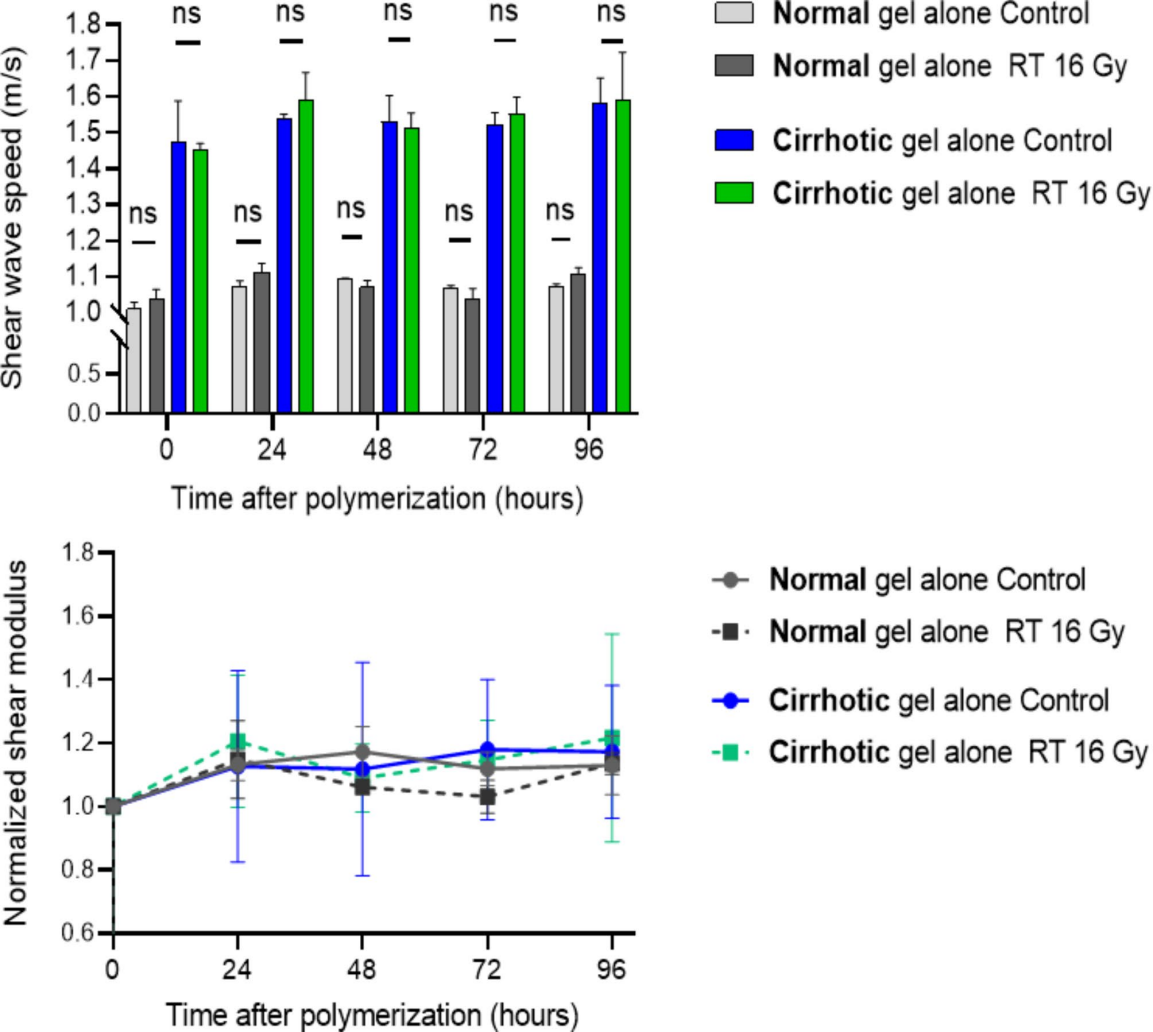
Shear wave speed (SWS) and normalized shear modulus of 3D gels of normal and cirrhotic stiffness without embedded cells, with and without irradiation (16 Gy). The top panel shows SWS values, while the bottom panel presents the normalized shear modulus over time. Data represent mean ± SD values from three independent experiments. “ns” indicates non-significant differences between sham and 16 Gy irradiation by independent t tests. Where multiple comparisons were made, the Šídák correction was applied to adjust for potential type I errors

Figure 3 illustrates the temporal alterations in the normalized shear modulus of gels for the normal and cirrhotic matrices. A notable increase in modulus after 24 h was observed in both groups, while the response to RT differed between the two groups. In the normal group, RT induced a statistically significant reduction in shear modulus compared to sham RT at 72 and 96 h. In contrast, there was no significant difference between the RT and sham control groups in the cirrhotic ECM at any time point. To validate the source of stiffness changes induced by RT, we created gel samples with the same composition but without embedded cells for comparison (Fig. 4). In line with our previous study [15], there was no statistically significant difference in SWS across all measurement time points in the 3D gels without embedded cells, regardless of whether they were in the normal or cirrhotic groups. Notably, the baseline stiffness of the gel-alone samples was much lower (average − 35.3%) than that of the cell-embedded cultures in either composition, highlighting that the cells themselves contribute significantly to tumor elasticity.

The results of the two-way repeated measures ANOVA for the longitudinal analysis of 3D sample stiffness for Figs. 3 and 4 are summarized in Supplementary Table 1 (Additional file1). RT demonstrated a significant effect on stiffness in normal cultures, while there was no significant impact in cirrhotic cultures or gel-alone samples. Interestingly, shear modulus changes significantly over time in the cirrhotic cultures, suggesting interactions between the cells and their surrounding matrix, given that RT had a lesser impact here.

### DNA damage and ECM stiffness

To ensure cellular integrity before biological assessments, various methods for isolating cells from the hydrogel were evaluated (Supplementary Tables 2 and Fig. 1, Additional file 1), with the most effective formulation identified as 0.9 U of dispase II and 100 U of collagenase type I per ml of calcium chloride solution, processed at 37 °C for 15 min. Initial cell viability was checked using trypan blue exclusion, and further cellular characteristics were assessed by flow cytometry. This formulation not only maximized viability but also consistently dissolved samples across different ECM stiffness levels, improving efficiency and reducing the need for extensive pipetting. This approach significantly enhanced cell recovery by minimizing mechanical stress on cells.

**Fig. 5:**
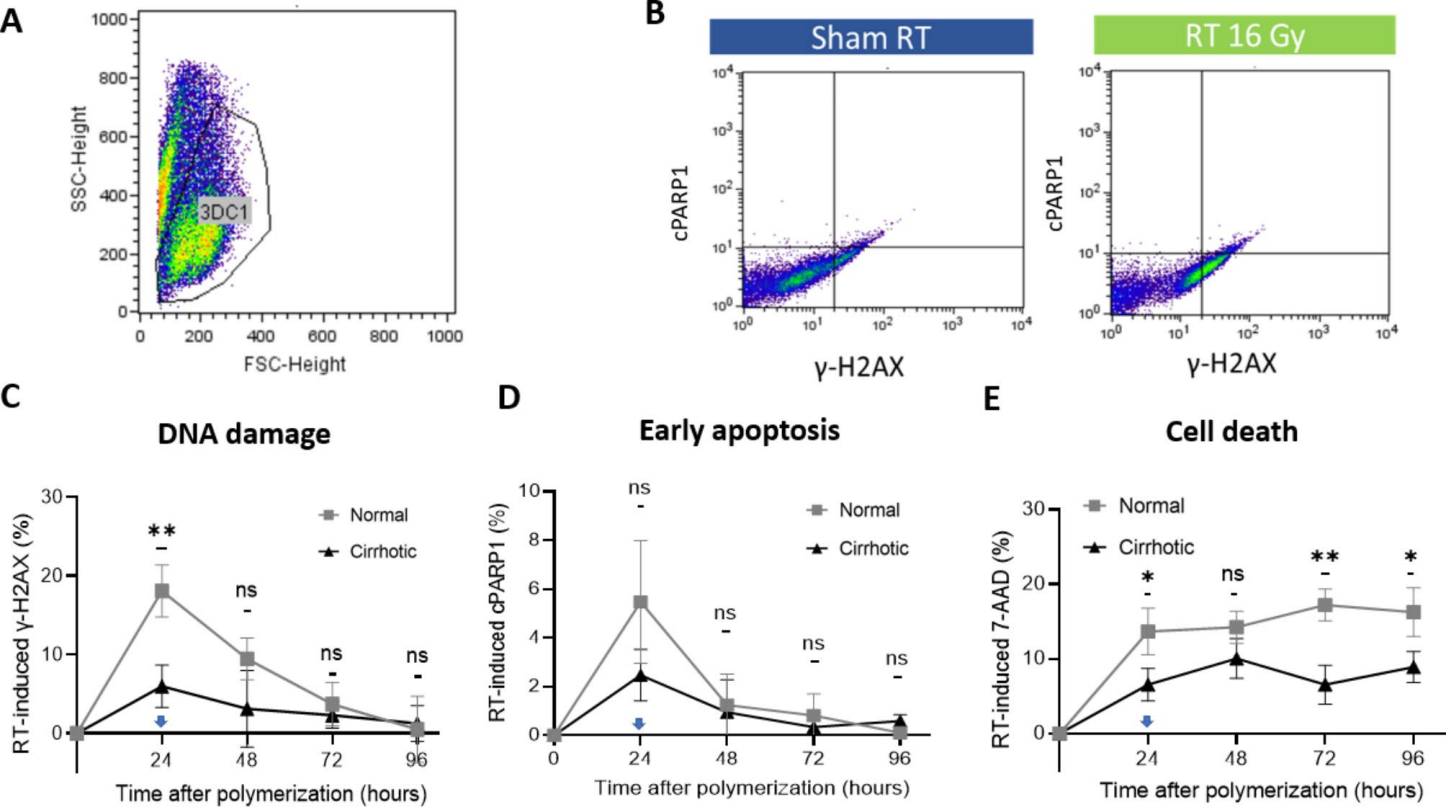
Flow cytometry plots and analysis. (**A**) A representative flow cytometry plot of the normal group. The forward scatter (FSC) and side scatter (SSC) parameters were utilized to differentiate the cell group of interest from residual matrix aggregates. The circled region labeled “3DC1” defines the cell group of interest for further analysis. (**B**) Demonstration of the gating for cleaved PARP1( cPARP1) and γ-H2AX in sham RT (left) and RT 16 Gy (right) arms for normal cultures at 24 h. Radiation-induced biomarkers of interests could be derived for further analysis. Temporal dynamics of radiation-induced γ-H2AX (**C**), cPARP1 (**D**), 7-aminoactinomycin D (7-AAD) (**E**) in cultures of different matrix. Arrows in panels C, D, and E indicate when RT of 16 Gy was applied. The flow cytometry assessments were conducted 2 h after RT and every 24 h along with the measurement of elasticity of the matrix. Data are mean and SD values from three independent experiments. * Indicates *p* < 0.05, ** indicates *p* < 0.01 and “ns” indicates non-significant differences between normal and cirrhotic cultures by independent t-tests after applying the Šídák correction for multiple comparisons at each time point

Following the isolation and recovery of cells, flow cytometry was used to assess DNA damage, early apoptosis, and cell death. Figure 5A shows a representative flow cytometry plot of normal group. The cell population of interest was identified based on their characteristic FSC and SSC profiles, which distinguish them from the larger, more granular aggregates typically seen in the matrix. Figure 5B demonstrates the gating for cPARP1 and γ-H2AX in both sham RT and RT 16 Gy of the normal matrix. In the sham RT group, the levels of γ-H2AX and cPARP1 remain low, indicating minimal DNA damage and cell death. In contrast, the RT 16 Gy group shows a significant increase in both γ-H2AX and cPARP1, reflecting higher DNA damage and apoptotic activity due to radiation treatment. We could then derive the radiation-induced γ-H2AX, cPARP1, and 7-AAD, as shown in Fig. 5C-E. Both the normal and cirrhotic cultures had the highest γ-H2AX signals 24 h after gel polymerization when RT was administered. In the normal group, γ-H2AX levels peaked at 24 h, significantly higher than in the cirrhotic group (*p* < 0.01), and decreased over time, indicating DNA damage repair processes. cPARP1 levels also peaked at 24 h in both groups, with no significant differences observed between normal and cirrhotic cultures at any time point (Fig. 5D.)

Figure 5E presents the staining intensity of 7-AAD, which assesses cell death at a later stage, based on the cell membrane permeability [23]. In the normal group, 7-AAD levels increased over time post-RT, with significant differences observed at 24, 72, and 96 h compared to the cirrhotic group. This indicates higher cell death in the normal group following radiation exposure. These findings underscore the influence of ECM stiffness on radiation response in a millimeter-sized 3D culture.

### LI for ECM stiffness alteration

**Fig. 6:**
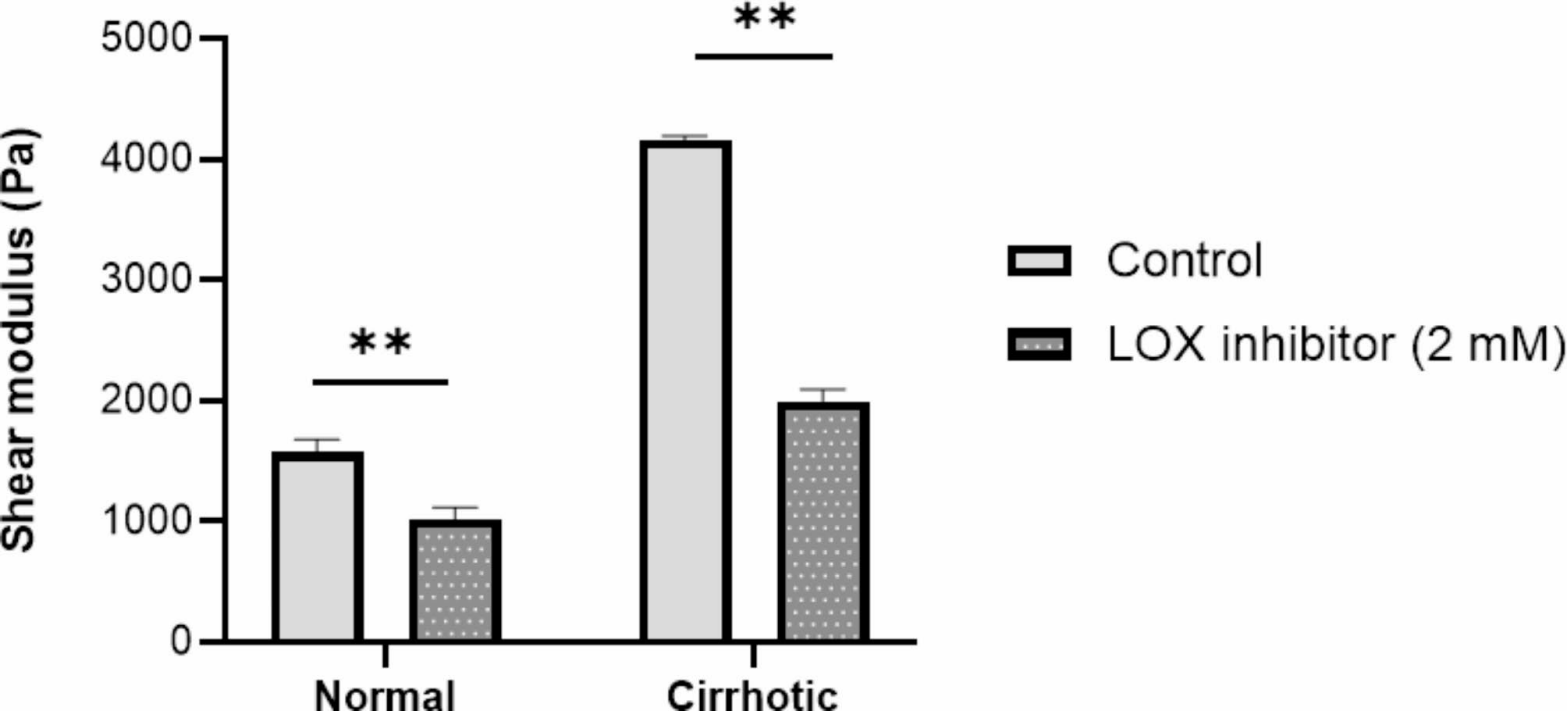
Shear modulus of normal and cirrhotic ECM with and without LOX inhibitor. A significant reduction in shear modulus was observed following LI with BAPN (2 mM). Data are mean and SD values from three independent experiments **, *p* < 0.01

The application of the LOX inhibitor, BAPN, resulted in a significant decrease in ECM stiffness for both the normal and cirrhotic matrices at the completion of polymerization (Fig. 6). The effect of LI on stiffness reduction was more pronounced in the cirrhotic than the normal ECM (mean reductions of 52.0% and 34.9%, *p* = 0.014).

**Fig. 7:**
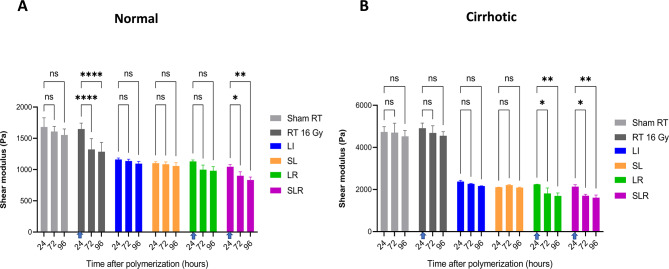
Dynamics of the absolute shear modulus in cultures with normal (**A**) and cirrhotic (**B**) stiffness. The shear modulus values are presented for time points at 24, 72, and 96 h after matrix polymerization completion. Radiotherapy (RT) of 16 Gy was applied at the 24-hour time point, as indicated by the arrows. LI: lysyl oxidase inhibitor, SL: combining sonoporation and LI, RT: radiotherapy, LR: combination of LI and RT, SLR: combination of sonoporation, LI, and RT. Data are presented as mean ± SD from three independent experiments. Statistical significance is indicated as follows: *p < 0.05, **p < 0.01, ***p < 0.001, ****p < 0.0001, and “ns” indicates non-significant differences by two-way ANOVA for multiple comparisons. The Dunnett test was used for multiple comparison correction with a family-wise alpha threshold of 0.05 for this presentation

Figure 7 illustrates the dynamics of the absolute shear modulus in cultures with normal (Fig. 7A) and cirrhotic (Fig. 7B) matrix stiffness following various treatment modalities. Given the significant decreases in normalized stiffness at 72 and 96 h previously highlighted in Fig. 3, the shear modulus values are presented at 24, 72, and 96 h after matrix polymerization completion. RT at a dose of 16 Gy was applied at the 24-hour time point, as indicated by the arrows in the figure. Direct comparisons between normalized shear modulus values could be misleading—especially considering that LOX inhibition significantly altered the baseline elasticity, as shown in Fig. 6. Therefore, we focused on presenting absolute shear modulus values to provide a clearer representation of the effects of different treatment modalities. In both normal and cirrhotic groups, LI alone or in combination with sonoporation (SL) showed no significant effect on shear modulus, despite the lower baseline stiffness compared to matrices without LI. In these matrices, the medium containing 2 mM BAPN was used throughout the incubation period to maintain LI. However, no further decreases in stiffness were observed in either group during the incubation period.

While RT at a dose of 16 Gy alone significantly decreased the shear modulus in the normal group (Fig. 7A), this reduction was not further enhanced by the combined treatment with LI (LR). In contrast, in the cirrhotic group (Fig. 7B), RT alone did not lead to a significant decrease in shear modulus at 72 and 96 h. However, the combination of RT with LI (LR) significantly reduced shear modulus at both 72 and 96 h. Additionally, in both groups, sonoporation combined with LI and RT (SLR) resulted in a significant reduction in stiffness at the timepoints of 72, and 96 h.

These results emphasize the critical role of RT in reducing matrix stiffness, particularly when combined with LI and sonoporation, highlighting the potential of these combined treatment strategies in modulating ECM stiffness, especially in cirrhotic environments. The Dunnett test was employed for multiple comparison correction, ensuring the robustness of these findings with a family-wise alpha threshold of 0.05.

### ECM stiffness and LI modulate DNA damage and cell death in response to RT

**Fig. 8:**
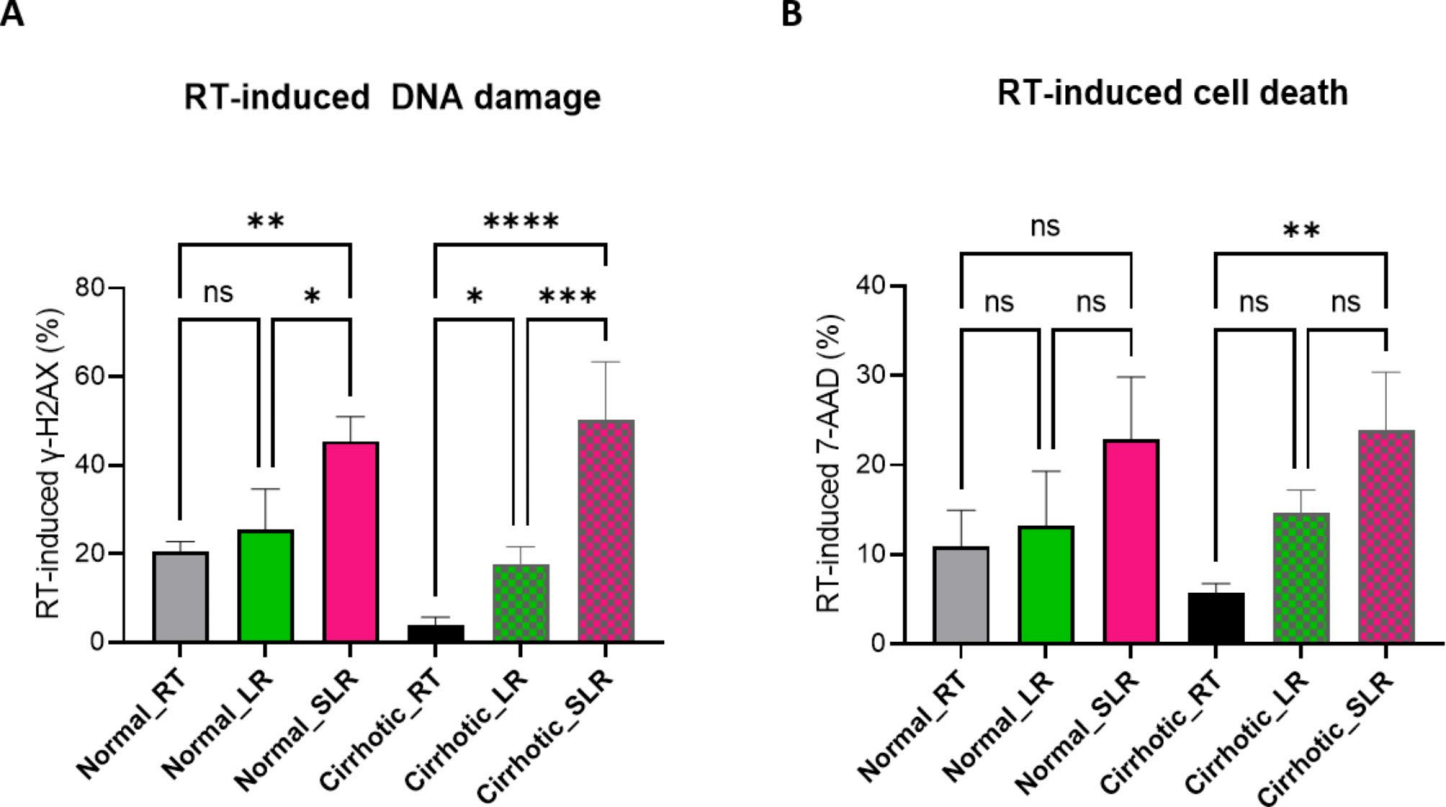
LI and Sonoporation-assisted LI Enhancement of RT-induced DNA Damage and Cell Death in Cirrhotic ECM. (**A**) RT-induced DNA damage as indicated by γ-H2AX levels. (**B**) RT-induced cell death as indicated by 7-AAD levels. Huh7 cells were cultured in normal or cirrhotic ECM and treated with RT alone, lysyl oxidase inhibitor and RT (LR), or the combination of sonoporation, lysyl oxidase inhibitor, and RT (SLR). γ-H2AX and 7-AAD levels were assessed at 24 and 48 h after gel polymerization, respectively. Data are presented as mean ± SD from three independent experiments. Statistical significance is indicated as follows: ns, not significant; *, *p* < 0.05; **, *p* < 0.01; ***, *p* < 0.001; ****, *p* < 0.0001 by ANOVA with Tuckey’s multiple comparisons test

Figure 8 compares RT-induced DNA damage, indicated by γ-H2AX staining intensity, and cell death, indicated by 7-AAD staining intensity (B), in Huh7 cells cultured in normal and cirrhotic ECM. γ-H2AX levels were assessed at 24 h after gel polymerization, while 7-AAD levels were evaluated at 48 h. Following RT, γ-H2AX level was notably lower in cells on the cirrhotic matrices than in those on the normal matrices, as shown in Fig. 5. However, when LI preceded RT (LR) γ-H2AX staining intensity significantly increased in the cirrhotic ECM, indicating heightened DNA damage. This increase was not observed in the normal ECM. These findings suggest that LI enhanced RT-induced DNA damage in a stiffness-dependent manner, with γ-H2AX level in the cirrhotic group with LI comparable to those in the normal group. Similarly, LR in the cirrhotic group resulted in significantly higher 7-AAD level compared to RT alone. In contrast this effect was not observed in the normal group, highlighting the role of LI in sensitizing cirrhotic ECM to RT-induced cell death.

### Enhanced radiosensitization by SLR

The combined approach of sonoporation-assisted LI before RT, SLR, significantly enhanced RT-induced γ-H2AX levels in both the normal and cirrhotic matrices. This enhancement was notably more pronounced in the cirrhotic group. Compared to LR, SLR further increased DNA damage in both groups (Fig. 8A). In terms of cell death, SLR significantly induced higher 7-AAD intensity than RT alone in the cirrhotic matrix. (Fig. 8B) While SLR did not produce more cell death than LR treatment in either group, there was a trend towards increased cell death, particularly evident in the cirrhotic matrix (cirrhotic SLR vs. LR *p* = 0.072).

## Discussion

This study was the first to investigate the impact of the TME stiffness on the radiation response of human liver cancer cells in a millimeter-sized, ECM-based 3D culture. The cultures were incubated for 24 h before RT was applied in situ. No additional cell resuspension was applied, in order to facilitate cell–ECM interactions. The dose of 16 Gy was empirically chosen based on our previous finding that a single dose of 16 Gy could delay Huh7 tumor regrowth [17]. Flow cytometry was used to assess the DNA damage and subsequent cell death after the cells were isolated from the hydrogels. This demonstrated that increased ECM stiffness was associated with higher radiation-induced effects of Huh7 liver cancer cells.

Liver cancer is one of the most-studied tumor entities in terms of ECM stiffness since this is often related to liver fibrosis [24]. Various studies have demonstrated that resistance to cisplatin, sorafenib, paclitaxel, 5FU, and oxaliplatin is affected by ECM stiffness [25–27]. Also, hepatocellular carcinoma cells cultivated in a high-stiffness matrix showed many stem-cell-like characteristics [20]. Although invasiveness and stemness are associated with radioresistance [28], the present study has demonstrated a direct association between ECM stiffness and radiation-induced DNA damage and cell death of liver cancer cells in a millimeter-sized 3D culture. Conventional studies employed hydrogels for incubating tumorspheres, and in that condition, the stiffness was predominantly determined by the underlying polyacrylamide background rather than the surrounding ECM [14]. In our millimeter-sized cultures, we ensure that most of the cells were enveloped by the ECM. A higher ratio of the ECM size to the size of the tumor aggregates creates a more isotropic microenvironment, particularly in terms of mechanosensing, which made our model more representative of the in vivo conditions. Moreover, by implementing SW-based elasticity measurement, we were able to assess the stiffness of the ECM in millimeter-sized 3D cultures. This novel method allowed us to assess the impact of TME stiffness on the radiosensitivity of human liver cancer under more realistic conditions.

It is essential to quantify the effect of radiosensitization [29]. There have been researches reporting cell survival in a 3D cell culture after RT with various approaches. Gomez-Roman et al. assessed the difference in radiosensitivity of GBM cancer cells between 2D and 3D environments [30]. The number of 3D colonies was manually counted. Similarly, the radiation response of head and neck cancer cell lines was investigated using 3D spheroid models, where clonogenic survival was assessed through phase-contrast light microscopy [31]. Another recent study measured the mean area of the spheroids to correlate the radiation response of HPV-positive head and neck cancer cell lines with 2D clonogenic assays​ [32]. These studies demonstrated that phase-contrast light microscopy can effectively observe spheroids or “colony numbers” in these smaller 3D systems.

However, we recognize that many 3D culture systems described in the literature, which often involve tumor spheroids embedded in a laminin-rich ECM of several tens to about one hundred micrometers, are primarily suited for analysis using light microscopy or plane-wave microscopy. In contrast, our model uses a gel that mimics the size of in vivo tumors, achieving a millimeter-scale size. This enhances our ability to study the tumor response to RT more realistically and to understand the mechanisms of treatment resistance. This advantage, however, restricts the use of direct observation of the tumor spheroid in situ. Therefore, we digest the millimeter-sized gel to recover the cancer cells and analyze their response to radiation via r-H2AX and 7AAD level instead.

The recovery of cells from 3D gels has also been reported [14], where the recovered cells were seeded on a 2D plate and then subjected to a traditional clonogenic assay​. However, we believe that transitioning cells from a 3D to a 2D microenvironment can drastically alter their behavior and therefore may not reflect survival in the original 3D culture. In the present study, after cell recovery, single-cell flow cytometry was employed to measure the γ-H2AX, cleaved PARP-1, and 7-AAD staining intensity 2 h after RT and every 24 h along with the measurement of elasticity of the matrix. While these markers do not directly measure clonogenic survival, which is the definitive test for long-term cellular recovery and proliferative capability after radiation exposure, they are indicative of immediate cellular responses. For instance, a study on intrinsic radiosensitivity in canine cancer cell lines linked residual γ-H2AX levels to outcomes in clonogenic assays [33]. We observed significant differences in γ-H2AX levels between the cirrhotic and normal groups 2 h post-RT. There was also a trend of difference at 24 h after RT (48 h after polymerization, *p* = 0.070). This delayed clearance of γ-H2AX in cells cultured in low-stiffness ECM aligns with findings that softer matrices can reduce DNA repair efficiency up to 24 h after 2-Gy irradiation [13]. These results highlight how ECM stiffness can influence DNA damage responses and, consequently, cell survival after RT. In our study, γ-H2AX levels were measured within 2 h post-RT, suggesting that while some repair processes might have begun, the predominant factor contributing to the observed peak would likely be the initial radiation-induced damage rather than after extensive repair. Despite variations in experimental variables such as culture size, matrix stiffness, and radiation dosage, we noted ongoing DNA repair activities 2–3 days post-RT in the group with normal matrix stiffness, underscoring the complex interplay between matrix properties and cellular DNA repair mechanisms.

In our study, the cPARP-1 levels showed a trend of difference between the normal and cirrhotic groups. While cPARP-1 is valuable for identifying early apoptosis, it does not capture the full extent of cell death, particularly late-stage apoptosis and necrosis. In contrast, 7-AAD in flow cytometry is effective for identifying necrotic or late apoptotic cells after treatment [34]. Therefore, we utilized 7-AAD as a more comprehensive marker for evaluating cell survival outcomes post-RT. However, apoptosis and necrosis represent just parts of radiation-induced cell death [33]. Our study did not assess cell cycle block, senescence [35], or autophagy [36]. Clonogenic survival assays measure the overall impact of various forms of cell death. Although our study did not include clonogenic survival assays, our platform effectively assessed RT-induced DNA damage and subsequent cell death within a millimeter-sized 3D culture. This innovative approach better simulates the liver cirrhosis environment, providing a significant advancement in studying the dynamic responses of cells to radiation in a setting that closely mimics in vivo conditions for the first time.

The temporal changes in stiffness following RT revealed intriguing dynamics within the normal and cirrhotic matrices. Previous studies found that tumor progression, whether in vitro or in vivo, was consistently accompanied by ECM stiffening [37–39]. This observation aligns with the initial increase in the shear modulus that we observed in both the normal and cirrhotic groups. Specifically, we consistently observed an increase in stiffness between 0 and 24 h after polymerization, across different stiffness levels and cell embedment conditions. Notably, the increase in stiffness is more apparent in cell-embedded cultures than in gel-alone samples. In addition, hydrogels composed of collagen fiber and Matrigel typically achieve complete polymerization within 30 to 60 min at 37 °C​​​ [40]. Although we could not prove whether there is ongoing polymerization between 0 and 24 h, it is more plausible that the observed increases in stiffness are predominantly due to cellular effects within the matrix. While RT led to a substantial reduction in shear modulus in the normal group, the cirrhotic ECM exhibited only a modest decrease. These observations suggest that the ECM in the cirrhotic background responds differently to RT, potentially contributing to radioresistance. Many clinical studies have found that the decrease in tumor stiffness following RT is an important predictor of the response to tumor treatment [41–43]. However, there have been few longitudinal observations of the stiffness changes in in vitro 3D tumor models after RT. Our previous study implied that the RT-induced change in ECM stiffness was due to interactions between cells and their surrounding ECM [15]. In the present study we went a step further to discover that the inherent elasticity of the ECM can affect how cells interact with the ECM after RT.

LOX is responsible for catalyzing the cross-linking of collagen and elastin within the ECM, and the expression of LOX family members is significantly upregulated in cirrhotic livers [6]. There is emerging evidence that LOX also influences cellular advancement and the metastatic potential of tumors [44, 45]. Previous research has found that inhibition or knockdown of LOX family members can help to reduce radioresistance in prostate cancer [45] and lung cancer [46]. In the present study, the induction of LI with BAPN produced an interesting dynamic, particularly in the cirrhotic ECM, where a pronounced decrease in stiffness was observed. This finding implies that targeting LOX activity might reduce the radioresistance of a stiff ECM, thereby enhancing the prospects for effective RT. The temporal dynamics of the stiffness for LR highlighted distinct responses between the normal and cirrhotic matrices, further demonstrating the complex interplay between ECM stiffness and treatment outcomes. It is important to note that while the application of the LOX inhibitor resulted in a significant initial decrease in ECM stiffness (as shown in Fig. 6), subsequent changes in stiffness were only observed when RT was applied. Specifically, the co-administration of RT with or without sonoporation (SLR and LR) led to a further decrease in matrix stiffness. In contrast, without RT, neither LI nor SL alone significantly decreased stiffness in either the normal or cirrhotic groups. This underscores the crucial role of RT in modulating ECM stiffness, particularly in the context of combined treatment strategies.

Our study also explored the concept of SL. Sonoporation involves the application of sound waves, typically in the ultrasonic range, to temporarily alter the permeability of cell plasma membranes, a technique that is utilized to improve the intracellular delivery of therapeutic agents and genetic materials [47]. The key to effective sonoporation lies in the induction of cavitation, a process that entails the formation, resonance, and subsequent destruction of MBs. This approach has proven effective in enhancing the delivery efficiency of gene therapy [47] and gold nanoparticles [17, 48]. Similarly, in the current study, we explored whether similar enhancements in therapeutic efficacy can be achieved with LOX inhibitors, using sonoporation to facilitate their delivery. Notably, the LOX inhibitor was delivered into Huh7 cells either with or without the aid of sonoporation before embedding these cells into the 3D gels. It’s crucial to note that in this setup, any cavitation effects were limited to the cell suspension phase and did not directly interact with the 3D matrix structure. Consequently, we did not observe cavitation impacts on the ECM in our experimental conditions.

There are several limitations to the proposed approach. First, while our integrated 3D culture platform incorporating SW-based elasticity measurements demonstrated the stiffness dynamics after RT and the effects of LI on radiosensitivity, further research is needed into the molecular pathways. There is emerging evidence that the crosstalk between DNA damage and ECM remodeling is not unidirectional [49], indicating a direct molecular connection between cell nuclei and ECM [50]. While we have demonstrated ECM remodeling after RT-induced DNA damage in a 3D culture, the detailed mechanisms that regulate ECM synthesis and degradation remain to be elucidated. Second, although the use of millimeter-sized samples within an isotropic microenvironment is closer to in vivo conditions, it must be acknowledged that cellular behaviors in a 3D culture may still differ from the complex, dynamic conditions in a living organism. The current platform does not include the interplay between cancer-associated fibroblasts and cancer cells, and so further investigations with a co-culture of fibroblasts or even an animal model is needed. Notably, the elasticity of the biomaterial was calculated by applying a time-of-flight algorithm to the reflected SWs from the side boundaries of the culture sample. Therefore, the stiffness of a tumor without such defined boundaries in live animals cannot be accurately probed using this platform. For these cases, SW elastography [51, 52] may be a more-suitable alternative for accurate stiffness assessments. Third, while our study utilized only the Huh7 cell line, which limits generalizability across various liver cancer types, it’s crucial to note that the stiffness of our 3D culture model was meticulously designed to mimic that of both healthy and cirrhotic human livers. This specificity ensures that our findings with the Huh7 cell line are highly relevant to human liver conditions, providing valuable insights into cellular behaviors in physiologically realistic environments. Further studies with different cell lines in animal models will help validate and extend our findings.

## Conclusions

A single-element ultrasound transducer platform for SW-based elasticity measurements has been applied to millimeter-sized, ECM-based 3D cultures of liver cancer cells. This noninvasive and technically straightforward approach has demonstrated that a stiffer ECM is associated with lower radiation-induced response. In addition to observing stiffness dynamics, we have developed a workflow to assess radiation response in millimeter-sized 3D cultures. Our investigations of LI have demonstrated its potential in modulating the ECM stiffness and enhancing treatment response. The combination of sonoporation with the LOX-targeting strategy further enhanced radiation response. Future deciphering of the underlying mechanisms is needed to develop a stiffness-targeted strategy for maximizing the therapeutic benefits of RT.

## Electronic supplementary material

Below is the link to the electronic supplementary material.

Supplementary Material 1

## Data Availability

No datasets were generated or analysed during the current study.
