# Supplementary material for: Evaluating ECM stiffness and liver cancer radiation response via shear-wave elasticity in 3D culture models
Source: Radiat Oncol. 2024 Sep 27;19:128. doi: 10.1186/s13014-024-02513-7 (PMC11430210; doi:10.1186/s13014-024-02513-7)

Supplementary Table 1: **Summary of statistical analysis for longitudinal dynamics of 3D sample stiffness**

The upper part of the table ("Normal" and "Cirrhotic") corresponds to the data presented in Figure 3, while the lower part ("Normal gel alone" and "Cirrhotic gel alone") refers to Figure 4. The significance of the row factor (time), column factor (treatment), and their interaction is analyzed using a two-way ANOVA. Specific time points showing significant differences in Šídák multiple comparisons test are also highlighted.

	RT factor	Time factor	Interaction	Significant time points (RT effect)
Normal	p = 0.004**	p = 0.065(ns)	p = 0.227(ns)	72 and 96 hours
Cirrhotic	p = 0.392(ns)	p = 0.015*	p = 0.470(ns)	None
Normal **gel alone**	p = 0.422 (ns)	p = 0.081(ns)	p = 0.422(ns)	None
Cirrhotic **gel alone**	p = 0.983 (ns)	p = 0.503 (ns)	p = 0.818 (ns)	None

*ns: not significant, *p < 0.05, ** p < 0.01

Cancer cell recovery from the 3D cultures

We observed that formulations combining both dispase and collagenase significantly reduced the need for pipetting to achieve suitable cell suspensions for flow cytometry, enhancing process efficiency and potentially reducing cell stress. Our results, standardized at 37°C for 15 minutes, show that a mix containing 0.9 U of dispase II and 100 U of collagenase type I per ml in calcium chloride solution yielded the highest cell viability with minimal variability. This effective formula consistently dissolved samples across various matrix stiffness levels, proving crucial for reproducible and reliable results in the 3D cultures.

Supplementary Table 2

Mean viability and cell recovery rates (%) from three repeated experiments using various enzymatic treatments in 3D cultures, presented as mean ± SD. Viability was assessed using trypan blue exclusion.

Trypsin (0.25%)	Dispase II	Collagenase typer I	Mean cell retrieval rate (%)	Mean Cell viability (%)
1 ml			51.7±21.0	83.0±2.9
1 ml		100U	84.4±21.0	86.8±2.5
1.2 ml		50U	82.7±10.6	90.3±1.2
	1.8U		59.4±21.9	78.5±3.0
	0.9U		56.4±14.8	84.3±4.0
		100U	76.7±14.8	85.2±3.2
		150U	67.5±22.9	85.0±1.0
	0.9U	100U	91.5±2.2	89.5±0.9
	0.9U	50U	85.6±5.9	87.2±1.6
	1.8U	50U	91.9±3.5	82.5±0.9
Cell suspension kit Minute™ (Invent Biotechnologies, USA)			22.9±10.6	29.5±9.3

Supplementary Figure 1 Retrieval and processing of cell-laden gels for flow cytometry analysis

The depicted sequence illustrates the retrieval process of cell-laden gels from the agarose support (left column), subsequent dissolution using collagenase/dispase (middle column), and mechanical dissociation with a pipette (right picture). The resulting cell suspension is then centrifuged for subsequent flow cytometry analysis.

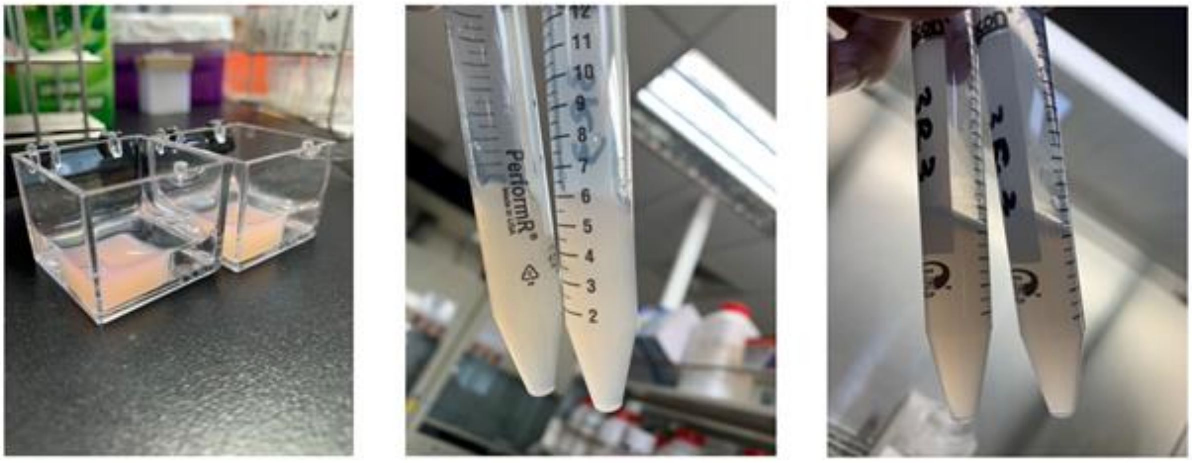

Supplement: Supplementary file 1 — Supplementary Material 1 [file 13014_2024_2513_MOESM1_ESM.pdf]
